# The Landscape of Pediatric High-Grade Gliomas: The Virtues and Pitfalls of Pre-Clinical Models

**DOI:** 10.3390/biology13060424

**Published:** 2024-06-07

**Authors:** Liam M. Furst, Enola M. Roussel, Ryan F. Leung, Ankita M. George, Sarah A. Best, James R. Whittle, Ron Firestein, Maree C. Faux, David D. Eisenstat

**Affiliations:** 1 Department of Paediatrics, University of Melbourne, Parkville, VIC 3052, Australia; liam.furst@mcri.edu.au (L.M.F.); enola.roussel@petermac.org (E.M.R.); ryan.leung@mcri.edu.au (R.F.L.); maree.faux@mcri.edu.au (M.C.F.); 2 Stem Cell Medicine, Murdoch Children’s Research Institute, Parkville, VIC 3052, Australia; ankita.george@mcri.edu.au; 3 Sir Peter MacCallum Department of Oncology, University of Melbourne, Peter MacCallum Cancer Centre, Melbourne, VIC 3000, Australia; whittle.j@wehi.edu.au; 4 Cancer Immunology Program, Peter MacCallum Cancer Centre, Melbourne, VIC 3000, Australia; 5 Personalised Oncology Division, The Walter and Eliza Hall Institute of Medical Research, Parkville, VIC 3010, Australia; best@wehi.edu.au; 6 Department of Medical Biology, University of Melbourne, Parkville, VIC 3010, Australia; 7 Department of Molecular and Translational Science, Monash University, Clayton, VIC 3168, Australia; ron.firestein@hudson.org.au; 8 Centre for Cancer Research, Hudson Institute of Medical Research, Clayton, VIC 3168, Australia; 9 Department of Surgery, University of Melbourne, Parkville, VIC 3010, Australia; 10 Children’s Cancer Centre, The Royal Children’s Hospital Melbourne, 50 Flemington Road, Parkville, VIC 3052, Australia

**Keywords:** pediatric high-grade gliomas, diffuse midline gliomas, diffuse hemispheric gliomas, histone H3K27 altered, histone H3G34 mutant, in vitro models, GEMM, organoids

## Abstract

**Simple Summary:**

Pediatric high-grade gliomas are aggressive and deadly brain tumors that arise in children and are notoriously difficult to cure. Researchers require accurate methods, or “model systems,” to better understand these tumors. Model systems include culturing cancer cells in a dish, observing how cancer cells grow in an animal brain, and growing cells in self-organizing 3D cultures called organoids, which more accurately resemble the brain. In this paper, we describe the molecular changes that these cancers acquire that cause them to be so aggressive, the development of past and present model systems, highlight the newest model systems being developed, and offer suggestions regarding how these model systems can be used in the future to develop better therapies for patients. Further, we outline the benefits and limitations of each model system to better guide researchers in the design of their experiments. Developing a variety of model systems that adequately and faithfully represent how tumors exist, progress, recur, and respond to treatment in a patient is absolutely essential to the eventual development of effective therapeutics. Therefore, investing in the continued development of these model systems is paramount to improving patient outcomes.

**Abstract:**

Pediatric high-grade gliomas (pHGG) are malignant and usually fatal central nervous system (CNS) WHO Grade 4 tumors. The majority of pHGG consist of diffuse midline gliomas (DMG), H3.3 or H3.1 K27 altered, or diffuse hemispheric gliomas (DHG) (H3.3 G34-mutant). Due to diffuse tumor infiltration of eloquent brain areas, especially for DMG, surgery has often been limited and chemotherapy has not been effective, leaving fractionated radiation to the involved field as the current standard of care. pHGG has only been classified as molecularly distinct from adult HGG since 2012 through Next-Generation sequencing approaches, which have shown pHGG to be epigenetically regulated and specific tumor sub-types to be representative of dysregulated differentiating cells. To translate discovery research into novel therapies, improved pre-clinical models that more adequately represent the tumor biology of pHGG are required. This review will summarize the molecular characteristics of different pHGG sub-types, with a specific focus on histone K27M mutations and the dysregulated gene expression profiles arising from these mutations. Current and emerging pre-clinical models for pHGG will be discussed, including commonly used patient-derived cell lines and in vivo modeling techniques, encompassing patient-derived xenograft murine models and genetically engineered mouse models (GEMMs). Lastly, emerging techniques to model CNS tumors within a human brain environment using brain organoids through co-culture will be explored. As models that more reliably represent pHGG continue to be developed, targetable biological and genetic vulnerabilities in the disease will be more rapidly identified, leading to better treatments and improved clinical outcomes.

## 1. Introduction

Brain cancers result in the greatest morbidity and mortality compared to other types of pediatric tumors [1,2,3]. Collectively, central nervous system (CNS) cancers are the most common solid tumor diagnosed in children [4] and on average result in a mortality rate of 0.66 children and adolescents per 100,000 population per year [5]. Broadly, intrinsic CNS tumors are classified into two major categories, glial or neuronal, according to their histopathologic, molecular, and prognostic characteristics [3]. These tumors are further classified into World Health Organization (WHO) grades based on several characteristics, including growth potential [6]. Pediatric-type diffuse high-grade gliomas (pHGG) represent a highly malignant subclass of CNS tumors, are grades 3 or 4 due to their aggressiveness and ability to invade brain tissue, and are associated with a very poor prognosis [6]. Within the pHGG tumor designation, there are four distinct sub-types defined by epigenetic and characteristic somatic mutations. The four classes that currently exist include: (1) Diffuse Midline Gliomas, H3 K27-altered; (2) Diffuse Hemispheric Glioma, H3 G34-mutant; (3) Diffuse pediatric-type high-grade glioma, H3-wildtype; and (4) IDH-wildtype, as well as infant-type hemispheric glioma [7,8]. Development of effective treatments against pHGG have universally failed. While the expected survival for all pHGG patients varies significantly between diagnoses, it does not typically exceed 5 years [9,10]. A major reason for the inability to develop effective therapeutics is the lack of biologically and clinically relevant pre-clinical models. This can be attributed, in part, to the rarity and frequently the neuroanatomic location of pHGG tumors, which often results in a paucity of available patient samples and limited biopsy tissue, especially for DMG. Until discoveries in the last decade distinguished pHGGs molecularly from adult high-grade glioma and resulted in distinct diagnoses and molecular subclasses, biopsy was often considered an unnecessary risk to patients with midline tumors, as it typically offered little to no clinical benefit. However, with neurosurgical and radiological advances, biopsy in the brainstem region is currently considered a relatively safe procedure [11] and is now more frequently offered to patients. In addition, post-mortem collection of tumor tissue has become more common [12]. In combination with advances in the availability of next-generation sequencing, this has allowed for a better understanding of the molecular drivers of pHGG and consequently, the continued development of pre-clinical models that more accurately represent patient tumors.

With the absence of knowledge regarding the different molecular drivers of pHGG and adult high-grade gliomas and the scarce availability of patient-derived tissue, earlier studies tended to rely on adult high-grade glioma-derived tissues [12]. However, in the past decade, due to an increase in the availability of patient tissue, pHGG is now considered an epigenetically dysregulated disease, with mutations in the tails of histone variants H3.1 and H3.3 driving malignancy and defining the two most common sub-types—diffuse midline gliomas (DMG) and diffuse hemispheric gliomas (DHG). Histone mutations lead to global changes in histone methylation and acetylation profiles, which leads to delayed or “stalled” differentiation and tumor stem cell properties.

This review will provide an overview of the sub-types of pHGG, as well as their driver mutations. The molecular effects of epigenetic dysregulation will be outlined. The outcome of this epigenetic dysregulation is represented by a stalled differentiation process, which itself varies depending on tumor subtype, thereby maintaining a cycling cell population within the tumor. As such, pHGG cell fate and biological characteristics will be discussed in detail. Finally, the development and uses of previous, current, and emerging pre-clinical models for the study of pHGG will be summarized, with a specific focus on the most prevalently modeled form of pHGG, DMG.

## 2. Pediatric-Type High-Grade Gliomas (pHGGs)

### 2.1. Diffuse Midline Glioma, H3 K27-Altered

**Figure 1:**
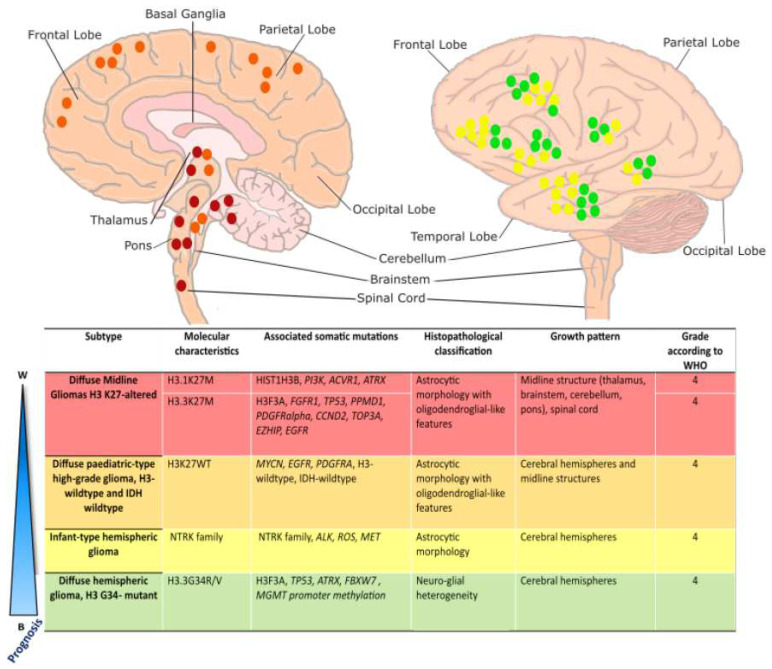
Overview of pediatric high-grade glioma sub-type localization: Schematic illustration of localization of pediatric high-grade gliomas, adapted from [17]. Diffuse Midline Gliomas H3 K27-altered tumors (red) most commonly arise in the pons, thalamus, and spinal cord and are driven by K27M mutations in histone variants 3.1 or 3.3. Diffuse pediatric-type high-grade gliomas, H3 wildtype, and IDH-wildtype (orange), lack characteristic histone mutations and may arise along the midline in either brainstem structures or the cerebral hemispheres. Infant-type hemispheric gliomas (yellow) arise superficially in the cerebral hemispheres and are characterized by variable RTK mutations. Diffuse Hemispheric Gliomas (green) arise superficially in the cerebral hemispheres and are driven by an H3.3 G34R/V mutation. DMG H3-altered, DMG H3-WT, and DHG G34R/V are all considered grade 4 tumors. Infant-type hemispheric gliomas are considered high-grade (grade 3 or 4).

First identified in 1926 by Harris and Newcomb and previously referred to as Diffuse Intrinsic Pontine Glioma (DIPG), DMG is the most common and deadly sub-type of pHGG [13]. The median age of diagnosis for patients with DMG tumors is 6–7 years, with an average survival of 9 months post-diagnosis, and approximately 90% of children do not survive 2 years post-diagnosis [14,15]. The clinical symptoms of DMG located in the pons or cerebellum (other neuroanatomic sites of DMG include the thalamus and spinal cord) are characterized by cerebellar signs such as ataxia, dysmetria, slurred speech (dysarthria), long tract signs such as increased muscle tone (hypertonia), over-reactive reflexes (hyperreflexia), as well as motor deficits such as hemiparesis. These are accompanied by cranial nerve palsies, which can be unilateral or bilateral and most commonly affect cranial nerves VI (affects eye movements) and VII (facial nerve) [16]. Children suffering from DMG display a progressive loss of control and coordination of the face, pharynx, and body [16]. Sequencing of a large cohort of patient tissue has shown a lysine to methionine mutation at codon 27 (K27M) in the histone 3 variant genes *H3F3A* and *HIST1H3B* to be the oncogenic drivers of greater than 80% of pHGG, most notably in DMG and DHG [17]. These genes encode the H3.3 and H3.1 histone variants [18], respectively, and further categorize DMG into H3.3K27M and H3.1K27M subtypes. DMG H3.3K27M is the most common form, occurring in 80% of DMGs, is more aggressive, and is associated with poorer outcomes compared to other DMG and pHGG subtypes (Figure 1). DMG H3.1K27M tumors have a slightly better prognosis, with a median survival of 15 months [19]. DMG H3.3K27M tumors occur in the pons, thalamus, cerebellum, and spinal cord, whereas DMG H3.1K27M tumors develop primarily in the pons [17] (Figure 1). These tumors are inoperable, disseminate, and invade surrounding structures such as the midbrain, medulla, cerebellum, thalamus, frontal cortex, and leptomeninges [20]. Although radiation in the involved field may provide disease stabilization and possibly extend both progression-free and overall survival by a few months, current treatments are ineffective, and the disease is almost uniformly fatal [21].

DMG H3K27M is characterized by epigenomic dysregulation due to the global reduction in repressive H3 K27 tri-methylation and global increase in activating H3 K27 acetylation. However, there may still be increased K27 trimethylation and decreased K27 acetylation at specific gene regulatory elements. This results in modified gene transcription in genes involved in neuronal proliferation, differentiation, function, and morphogenesis [22,23]. Due to aberrant differentiation, DMG cells retain an oligodendrocyte precursor cell (OPC)-like cell fate [24,25,26]. In DMG H3.3 K27M, secondary mutations arise in tumor suppressor genes (such as TP53) growth factor receptors (i.e., PDGRFα, EGFR, and FGFR1α), in addition to factors involved in development and cell cycle regulation (i.e., CCND2). In contrast, DMG H3.1K27M tumors harbor additional secondary mutations in PI3K, ATRX, and ACVR1 (Figure 1).

### 2.2. Diffuse Hemispheric Glioma, H3 G34-Mutant

Diffuse hemispheric gliomas H3.3 G34-mutant are characterized by the substitution of a glycine to an arginine or valine at residue 34 (G34R/V) in histone variant H3.3 [7] (Figure 1). This type of pHGG is almost completely restricted to the cerebral hemispheres and is mostly found in adolescents and young adults [27]. Patients have a longer overall survival compared to other H3-mutant groups (overall, 22 months post-diagnosis), although the prognosis for survival remains poor compared to most other childhood brain tumors [17]. The G34R/V point mutation in H3F3A co-segregates with TP53 and ATRX mutations and is frequently associated with MGMT promoter methylation, which is a strong prognostic factor regarding the outcome of adult patients with glioblastoma (GBM) [17,28]. The cell of origin of DHG is likely a GABAergic interneuron-like cell [29]. The role of H3.3G34 mutations in pHGG tumorigenesis requires further investigation [30].

### 2.3. Diffuse Pediatric-Type High-Grade Glioma, H3-Wildtype and IDH Wildtype

pHGGs that lack H3 and IDH1 mutations, called diffuse pediatric-type HGG, H3-wildtype, and IDH-wildtype (wt), are part of a heterogeneous group of tumors that contain distinct subgroups of genomic and epigenetic profiles with variable clinical behavior [7]. This type of tumor generally develops in children between 3 to 5 years old [31] and typically arises in the cerebral hemispheres and midline structures (Figure 1). The clinical outcome can vary according to the type of tumor [17], as mutations occur in varied RTK genes, including *EGFR*, *PDGFRα,* and *MYCN* [32,33].

### 2.4. Infant-Type Diffuse Hemispheric Glioma

Infant-type diffuse hemispheric glioma is a recent subtype included in the 2021 WHO classification of CNS tumors [7]. Unlike other pHGGs, these tumors are not immediately considered grade 3 or 4 tumors; however, they are classified as “high-grade” due to 5-year survival rates ranging from 25–50%. They have been characterized by frequent fusion mutations in receptor-tyrosine kinases (RTK), and as such, treatment may be personalized to individual patients through inhibition of specific signaling pathways [32,34].

pHGG, H3- and IDH-wt, and Infant-type DHG subtypes are reviewed in [32,35,36] and will not be discussed further here. We will primarily focus on H3 K27M mutations in DMG and the resulting epigenetic dysregulation of these tumors.

### 2.5. Epigenetic Alterations Caused by Histone Mutations

#### Global Epigenetic Dysregulation in pHGGs

**Figure 2:**
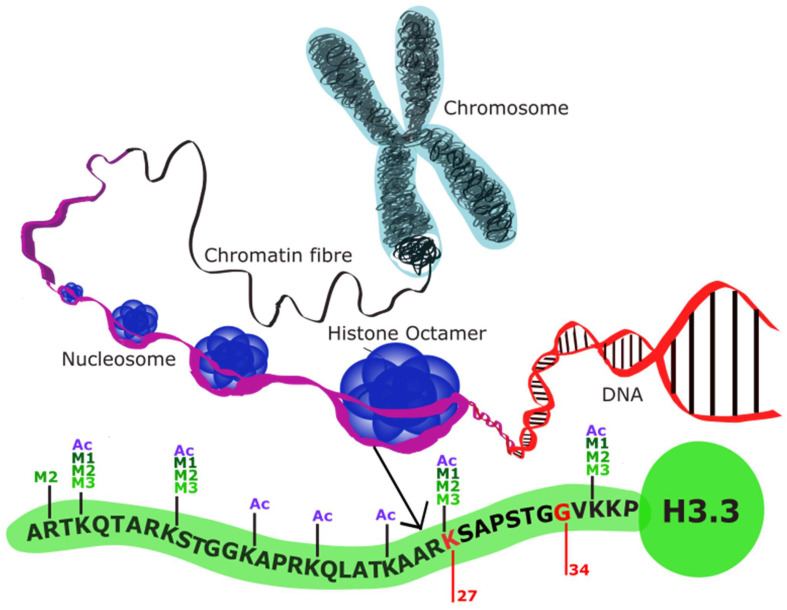
Histone structure (adapted from [18]): Schematic demonstrating the structure of histone octamers. The structure of histone variant H3.3 is shown here as a representative of the histone variants altered in pHGG. Genes encoding K27 and G34 are mutated in DMG and DHG, respectively, preventing histone methylation at the N-terminus.

In the preceding decade, various studies have established histone mutations as the molecular driver behind pHGGs; namely K27M substitutions in DMGs and G34 mutations in DHG [19,37]. These mutations are considered to interfere with the post-translational modifications (PTMs) that are applied to the tail of the histone variants and consequently result in large-scale epigenetic dysregulation within the cell. Lysine and arginine residues located on the N-terminal histone tails can be modified post-transcriptionally by many PTMs, especially acetylation, and mono-, di-, or trimethylation, which ultimately alters chromatin structure and regulates gene transcription (Figure 2).

In DMG, various studies have shown that the K27M mutations in the genes *H3F3A* and *HIST1H3B* result in an inability for the trimethylation repression marks to be deposited, and concurrently, there are significant downstream transcriptional alterations [19,22,37,38]. Notably, H3.3K27M DMG displays greater transcriptional dysregulation due to the increased presence of the H3.3 histone variant when compared to the H3.1 histone variant at high transcriptional activity sites, as the core histone H3.1 is scattered evenly through the genome [39,40]. Several studies have reported that the K27M mutation acts as a gain-of-function mutation with the capacity to disrupt the interaction between Polycomb Repressive Complex 2 (PRC2) [22,41,42]. PRC2 is composed of 3 subunits: EZH2, SUZ12, and EED. EZH2 and SUZ12 catalyze the trimethylation of Lys27 on histone 3 variants [37], which is heavily associated with inactive gene promoters. When present, K27M appears to bind and thereby restrict the enzymatic activity of EZH2, ultimately resulting in a failure of PRC2 to limit the spread of H3K27me3 and leading to downstream transcriptional dysregulation.

There have been various contradictory reports of changes to global histone 3 acetylation (H3K27ac), a marker associated with active transcription, in DMGs. Some studies have reported unchanged acetylation profiles in DMG cells [41,43,44], whereas others have reported retained or gained H3K27ac in H3.3K27M DMG cells [42,45]. One study showed that H3K27M cells displayed not only a global decrease in H3K27me3, but also a global increase of H3K27ac on H3.1/H3.1/H3.3 nucleosomes, with these depositions being significantly altered relative to H3K27WT or *IDH1* mutated tumors [46].

Less is known about the mechanism by which H3G34R/V mutations alter the global epigenetic profile; however, it is believed that the change to G34 impacts trimethylation of the nearby K36 residue by inhibiting the catalytic activity of the histone methylase SETD2 [47,48]. Loss of SETD2-mediated trimethylation has been associated with inhibition of neuronal activity and proper differentiation in other diseases and would therefore be consistent with the altered differentiation state seen in DHG [49,50].

**Figure 3:**
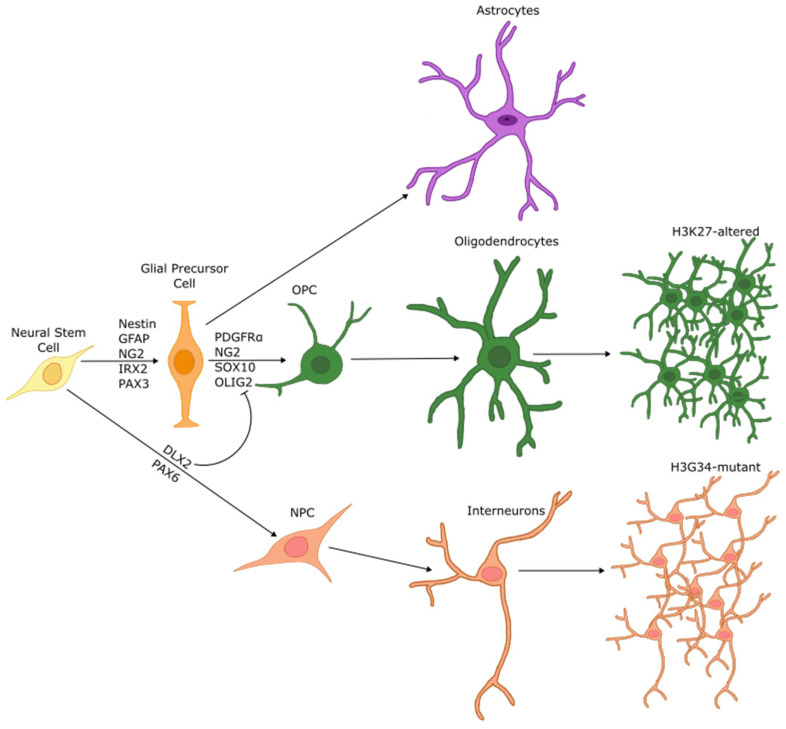
Differentiation of NSCs is halted during pHGG pathogenesis: Schematic describing the cell of origin for DMG and DHG. In a non-tumor state NSCs are differentiated into astrocytes, oligodendrocytes, or neurons. In DMG, H3 K27M mutations result in a failure to properly differentiate, resulting in high expression of OPC markers such as OLIG2, PDGFRα, SOX10, and NG2, implicating an OPC cell of origin. Conversely, G34R/V DHG tumors are halted during differentiation towards a GABAergic interneuron cell fate, with high expression of DLX2 and PAX6 transcription factors.

Ultimately, histone mutations result in a globally dysregulated epigenome. The inability to properly regulate the differentiation transcriptional programs results in stalled differentiation, which contributes to a highly oncogenic state. Using single cell RNA sequencing and advanced bioinformatics platforms, Monje et al. [51] and Filbin et al. [24] reported the cell of origin of DMG to be an OPC since the expression of transcription factors crucial for oligodendrocyte specification is upregulated in 80% of DMG cases [24]. Later analysis has shown that an OPC-like cell transcriptional state is common among most DMG subtypes, regardless of the anatomic region of the brain from which it arises or the age of the patient. [25]. Further analysis has shown that H3.1 and H3.3 tumors are characterized by distinct and different OPC lineage development programs [26], suggesting that while these tumors resemble one another, they arise from different points in OPC development (Figure 3).

## 3. In Vitro and In Vivo Pre-Clinical Models for the Study of pHGG

### 3.1. In Vitro Patient-Derived Cell Line Models for pHGG (Table 1)

The generation of in vitro models for pHGG has long been hindered by a lack of patient tissue, especially for DMG. Due to both the location and diffuse growth pattern in which DMG arises, surgical resection is not routinely part of the standard of care, and biopsy of the tumor was historically considered to be high-risk while offering little to no clinical benefit to patients [52]. However, following the increased availability of advanced sequencing technologies and the discoveries of histone driver mutations discussed in the preceding sections that have distinguished pHGGs from adult glioblastoma, increased importance has been placed on tissue collection through biopsy and post-mortem autopsy. As such, recent studies have reported that stereotactic biopsy of DMG can be safely performed [11,53,54]. In tandem with the increased importance placed on the retrieval of tumor tissue at autopsy, the availability of patient tissue has increased dramatically in the last decade, and with it, protocols for the efficient generation of patient-derived cell lines [12]. Modern cell culture for DMG uses a culture medium that resembles neural stem cell media, with Neurobasal A, DMEM/F12, supplemented with PDGFRAA, PDGFRBB, basic FGF, EGF, and heparin. DMG cell cultures grow as both spheroids and adherent cells when cultured on normal tissue culture treated plates [51]. The increase in the availability of these patient-derived tumor cell lines and tissues has contributed to many of the major discoveries regarding pHGG in the last decade [55,56], most notably in cell of origin research [24], but it is also enabling high-throughput investigation of cancer cell vulnerabilities in translational research through the use of CRISPR functional genomic screens [57,58,59,60] and medium- to high-throughput drug screens [61,62,63]. As with many other cancer cell line models, the ability of cells grown in culture in vitro to accurately represent the patient’s tumor is limited. Loss of genetic heterogeneity is common in cancer cell lines that have been passaged for a long period of time [64]. Likewise, monoculture of cancer cells fails to consider various environmental constraints that persist in tumors, such as hypoxia [65], extracellular matrix (ECM) changes [66], angiogenesis [67], or myeloid cell infiltration [68], which may all play a part in increasing tumorigenesis.

**Table 1:** pHGG cell lines and their associated co-mutations.

Cell Line	Histone Status	Mutational Status	Reference
PB19SH058	H3.3K27M	TP53 V157F, H3K27M	[60]
CNMC_D_874	H3.3K27M	NA	[60]
VUMC-DIPG-8	H3.3K27M	H3K27M	[60]
SU-DIPG-13	H3.3K27M	TP53 mutated, H3K27M	[55,56,63]
SU-DIPG-17	H3.3K27M	H3K27M	[55]
SU-DIPG-19	H3.3K27M	H3K27M	[55]
SU-DIPG-25	H3.3K27M	H3K27M	[55]
SU-DIPG-27	H3.3K27M	H3K27M	[55]
SU-DIPG-29	H3.3K27M	H3K27M	[55]
SU-DIPG-35	H3.3K27M	PPM1D S432 *, H3K27M	[56]
SU-DIPG-6	H3.3K27M	TP53 mutated, H3K27M	[55,56,69]
SU-DIPG-24	H3.3K27M	H3K27M	[55]
7316_388_A	H3.3K27M	TP53 R248W, PDGFRA D842V, H3K27M	[70]
7316_3058_S	H3.3K27M	PIK3CA E542K, TP53 R273H, A276T, R283C CDKN2A D74A, H3K27M	[70]
7316_1763_S1	H3.3K27M	PIK3CA E542K, TP53 R273H, A276T, R283C CDKN2A D74A, H3K27M	[70]
SU-pSCG1	H3.3K27M	NA	[55]
P005306	H3.3K27M	ATM R1466 *, PIK3R1 F456Q, H3K27M	[60]
BT245	H3.3K27M	H3K27M	[71]
VUMC-DIPG-11	H3.3K27M	H3K27M	[72]
VUMC-DIPG-A	H3.3K27M	H3K27M	[63,73]
HGG080318	H3.3K27M	H3K27M	[60]
7316_6349_S	H3.3K27M	TP53 R175H, C141Y; PDGFRA N659K, H3K27M	[70]
JHH-DIPG-1	H3.3K27M	H3K27M	[63,74]
SF7761	H3.3K27M	PPM1D E540 *, H3K27M	[75,76]
JHH_DIPG_2J	H3.3K27M	NA	[60]
P002306	H3.3K27M	ACVR1 G328E; PPM1D W427*; PIK3CA H1047R, H3K27M	[60]
SF8628	H3.3K27M	H3K27M	[73]
P001401	H3.3K27M	TP53 R175H, BRCA2 M3181fs, H3K27M	[60]
7316_195_S	H3.3K27M	TP53 X331splice, H3K27M	[70]
HSJD-DIPG-007	H3.3K27M	ACVR1 R206H; PPM1D P428Qfs *, H3K27M	[56,77,78]
7316_6475_S1	H3.3K27M	NRAS Q61K; FBXW7 X195splice; RB1 X474splice, H3K27M	[70]
7316_1769_S	H3.3K27M	PIK3CA Q546H; PTPN11 A72T; PIK3CA T1025A, H3K27M	[70]
PBT-29FHTC	H3.3K27M	FGFR1 546K; PIK3CA R93P; TP53 S127P, H3K27M	[60]
PBT-22FHTC	H3.3K27M	TP53 R306*, H3K27M	[60]
PPMP058_140222	H3.3K27M	TP53 R158L, H3K27M	[60]
ICR_B169_2D	H3.3K27M	TP53 C176Y; BRAF G469V, H3K27M	[60]
ICR_B181_2D	H3.3K27M	TP53 R273H; ACVR1 G328E; PIK3R1 N564D, H3K27M	[60]
RA055	H3.3K27M	TP53 R175H; PDGFRA amp, H3K27M	[60]
SU-DIPG-21	H3.1K27M	H3K27M	[55]
SU-DIPG-33	H3.1K27M	H3K27M	[79]
SU-DIPG-36	H3.1K27M	H3K27M	[79]
SU-DIPG-4	H3.1K27M	ACVR1 G328V, H3K27M	[55,63]
SU-DIPG-38	H3.1K27M	H3K27M	[79]
P003302	H3.1K27M	TP53 R273C; ACVR1 R206H, H3K27M	[60]
P005401	H3.1K27M	TP53 Arg306 *, H214Glnfs *; EGFR A289S, A289V, H3K27M	[60]
UON_JUMP4	H3.1K27M	TP53 R273H; PIK3CA E545K; ACVR1 G328V, H3K27M	[60]
ICR_B184_2D	H3.1K27M	TP53 C275Y; PIK3CA E542K, H3K27M	[60]
ICR_B301_2D	H3.1K27M	TP53 C135F; ACVR1 G328V; BCOR E858fs, H3K27M	[60]
OPBG_DIPG_004_2D	H3.1K27M	TP53 R348Q; ACVR1 G328E, H3K27M	[60]
DUB_D003_2D	H3.1K27M	TP53 R175H; KDM6B R513P, H3K27M	[60]
RCH4065	H3G34	PTEN del; CDKN2A del; PDGFRA amp, H3G34R	[60]
KNS_42	H3G34	BLM S186 *; TP53 R342 *, H3G34R	[80]
HSJD_GBM_002	H3G34	TP53 R209fs; NF1 L1246fs; CDKN2A/B del, H3G34R	[60]
ICR_CXJ_046	H3G34	H3G34R	[60]
OPBG_GBM_001	H3G34	TP53 mutated; PDGFRA Y288C; PTEN F341V; ATRX mutated, H3G34R	[60]
7316_158_S	H3G34	TP53 C238W, P152L, H3G34R	[70]
CNMC_760_XD	H3 WT	NA	[60]
VUMC-DIPG-10	H3 WT	NF1 Q209 *; MYCN amp	[60]
7316_913_S	H3 WT	PDGFRA N848K	[70]
ICR_B194_2D	H3 WT	TP53 R158fs	[60]
7316_5335_S2	H3 WT	KRAS G12V; CTNNB1 S33Y; EGFR N771_H773 dup; TP53 R342P	[70]
7316_5335_S1	H3 WT	KRAS G12V; CTNNB1 S33Y; EGFR N771_H773 dup; TP53 R342P	[70]
7316_1746_S	H3 WT	NA	[60,70]
P007401	H3 WT	TP53 G245S; RB1 R579 *; NF1 R1949Sfs; PTEN R173H; MSH6 R300W	[60]

* denotes common notation used for termination, i.e., a nonsense mutation induced early stop codon.

### 3.2. Modeling pHGG In Vivo Using Mice (Table 2)

#### 3.2.1. Genetically Engineered Mouse Models for pHGG

Genetically engineered mouse models (GEMMs) are an effective tool for understanding tumor biology initiated by a specific combination of mutations and in doing so, provide preliminary evidence towards the developmental cell of origin. In the context of pHGG, these models have been very useful in deciphering the specific developmental lineages being altered during tumor initiation by targeting specific cell types with tumor-initiating mutations.

**Table 2:** Summary of murine models used to represent pHGG.

Model	Cell Target	Molecular Alterations	Tumor Formation Latency	Result	Reference
GEMM	Nestin+	PDGF-B overexpression, Ink4a loss RCAS-PDGFB; Ntv-a; Ink4a-ARF −/−	4 weeks	PDGF-B overexpression alone caused low-grade tumors; PDGF-B overexpression and Ink4a loss caused high-grade tumors	[81]
	Nestin+	H3.3K27M overexpression, p53 loss RCAS-PDGF-B, RCAS-Cre, ±RCAS-H3.3K27M in nestin tv-a; p53^fl/fl^	Not disclosed	Not sufficient to generate gliomas, but did generate proliferating clusters	[42]
	Pax3+	PDGF-B overexpression, p53 loss, H3.3K27M overexpression Pax3-Tv-a; p53^fl/fl^, with RCAS-PDGF-B + RCAS-Cre or RCAS-PDGF-B + RCAS-Cre + RCAS-H3.3K27M	34–83 days	Sufficient to create high-grade, Olig2+ tumors, but tumors are not localized to the brainstem	[82]
	Olig2+	PDGF-B overexpression, p53 loss, H3K27M overexpression Olig2-Tv-a-Cre; p53^fl/fl^, and Olig2-Tv-a-Cre; p53^fl/+^ Olig2-Tv-a-Cre; PDGF-A; H3.3K27M Olig2-Tv-a-Cre PDGF-B; H3.3K27M Nestin-Tv-a; p53^fl/fl^	Not disclosed	Formed tumors, but was considered inadequate compared to Nestin+ GEMM	[83]
	Acvr1+	Acvr1 G328V, H3.3K27M, Pik3ca mutations Acvr1^floxG328V/+^; Hist1h3b^K27M/+^; Pik3ca^floxH1047R/+^; Olig2^Cre/+^	Neurological symptoms begin shortly after birth, mice survive for 419 days	Formed tumors, arrested cell lineage in the pre-OPC cell state.	[84]
	NSCs	H3.3K27M/PPM1DΔC/PIK3CAE545K, H3.1K27M/ACVR1G328V/PIK3CAE545K, H3.3K27M/p53LOF/FGFR1N457K, H3.3K27M/NF1LOF/FGFR1N457K, H3.3K27M/p53LOF/CCND2WT, H3.3/1 K27M with p53 LOF, or NF1LOF, or FGFR1N457K	20–60 days	Tumors develop in the brainstem proper	[85]
	Engraftment cell	Animal model	Result	Reference	
Brainstem Engraftment-based	Adult rat glioma C6, F98, 9L	Rat, adult and neonate	Successful engraftment in the brainstem, tumors histologically resembled DMG but not molecularly	[86,87,88,89,90]	
	Adult GBM cell lines (U87 MG, U251, GBM6, GBM14)	Athymic mice	Successful engraftment in the brainstem, tumors histologically resembled DMG but not molecularly	[91]	
	GBM primary patient tumor	NOD-SCID mice	Successful engraftment in the brainstem, tumors histologically resembled DMG but not molecularly	[92]	
	DMG primary patient tumor	NOD-SCID mice	Successful engraftment, tumors molecularly retain patient characteristics	[51,69,93]	
	Model system	Culture technique	Result	Reference	
Tumor organoid models	GBM, “organ-on-a-chip”	Printed with decellularized ECM	GBM organoids are able to invade and proliferate through ECM	[94]	
	3D GBM organoid	Patient-derived organoids suspended in Matrigel, cultured on a shaking plate	Organoids recapitulate hypoxia gradients; organoids were long-lived	[95,96,97,98]	
Organoid co-culture	GBM-cerebral organoid co-culture	GBM cells co-cultured with cerebral organoids (representative of a developing brain)	GBM cells successfully invade and proliferate within the cerebral organoid and retain the patient phenotype	[99,100]	
	DMG cortical organoid co-culture	DMG cells co-cultured with printed organoid	DMG cells are adequately targeted by HDAC inhibitors, showing similar results to previous pre-clinical data	[101]	

The earliest GEMM modeling brainstem glioma was generated using the chicken retroviral RCAS/tv-a system to concomitantly overexpress PDGF-B with loss of p16/Ink4a-p14/ARF (CDKN2A) in Nestin-expressing progenitor cells [81]. This resulted in high-grade, diffuse glioma-like tumors that occupied large portions of the pons with diffuse invasion into the surrounding brain structures [81]. Using this model, Becher et al. [81] also identified that the cells that led to tumors preferentially expressed *Olig2*, as opposed to expressing astrocytic differentiation markers. The establishment of this method also allowed for lineage tracing of the infected cells. Using an RCAS/GFP construct, the authors found that *Nestin*+ cells were located primarily in the outer layer of the 4th ventricle [81]. This model was used in a future study that identified PRC2 as an epigenetic catalyst for global histone acetylation in DMG and established that transgenic H3.3K27M was sufficient to increase global acetylation in *Nestin+* progenitor cells [42].

Misuraca et al. [82] built on this earlier mouse model using the RCAS/tv-a system with *Pax3+* cells instead of *Nestin+* cells. This was performed to better identify a potential cell of origin for DMG, as *Nestin* is expressed through a multitude of progenitor cell populations within the brainstem at multiple time points. *Pax3* is expressed in more specific cell populations and in specific combinations with other stem markers. This model showed that injection of RCAS-PDGF-B was sufficient for the growth of low-grade tumors. When driven by PDGF in *p53*-deficient cells, high-grade tumors formed, and the addition of RCAS-H3.3K27M led to a reduction in global methylation, more accurately representing patient DMG. Targeting *Pax3+* cells engages PDGF signaling, resulting in high levels of *Olig2* expression in these tumors. However, these tumors grew in various regions of the brainstem, thereby not accurately representing the majority of DMG tumors arising in the pons [82].

A further version of this GEMM was developed, targeting *Olig2*+ cells with PDGF-A or PDGF-B and H3.3K27M [83]. Interestingly, mouse survival was not significantly affected when compared to an H3.3WT model. Additionally, when compared to a model targeting *Nestin*+ cells, more *Nestin*+/H3K27M+ cells were observed than *Olig2*+/H3K27M+ cells. This suggested that although the cell of origin for DMG may share characteristics with OPCs, it is more likely to represent an earlier precursor cell. These results also speak to the effectiveness of GEMMs in probing the developmental origins of cancers such as DMG. This finding was in part supported by another GEMM created by Fortin et al. [84], who induced an *Acvr1* mutation (G328V) as well as *Hist1h3b* (K27M) and *Pik3ca* mutations to model the mutational landscape seen in H3.1K27M tumors. These mutations were sufficient to arrest cells in the OPC lineage with increased PDGFRA expression, leading to tumor formation. *Acvr1* is commonly mutated in DMG with the H3.1K27M mutation and is a marker of an earlier lineage NPC, supporting the notion that the oncogenic potential of H3K27 mutations acts on an earlier cell lineage than OPCs and results in cell fate arrest along the OPC lineage [84].

As the networks formed between neurons and tumors are shown to be increasingly important to tumor progression [102,103,104], optogenetic GEMMs may become a more frequently used technique to investigate DMG. Optogenetic models allow for the activation of specific neural circuits using light-gated ion channels called channelrhodopsins [105]. Studies into how a specific neural circuit can promote tumor growth using this method may open opportunities for treatments that modulate specific neurotransmitters. As tumors that arise in GEMMs are derived from autochthonous cells, they will similarly form or have existing neuron-tumor interactions. Optogenetic models may therefore have increasing importance in studies using GEMMs, with the ability to better study these intercellular interactions.

A more recent study was able to generate a multitude of mouse models using in utero electroporation (IUE) of H3 mutations with candidate partner mutations into mouse embryos. These models were able to evaluate the H3.3 G34R mutation with its associated mutation in the *PDGFRA* gene, showing that G34R DHGs can arise from the ganglionic eminences (similar to the germinal matrices of human embryos), as well as generating mouse models for H3K27M with mutations in *NF1, FGFR1, PPM1D, PIK3CA*, and *ACVR1*. In addition to targeting these cancers in a spatiotemporal context, models incorporating IUE methods allow for the evaluation of these cancers of development to be investigated in the developing brain, which contrasts to previous models in which tumors grow in adult mice [85].

While GEMMs are, without dispute, an important tool for understanding pHGG pathogenesis, they also have some inherent caveats. First, all GEMMs are created using a limited number or specific set of pathogenic mutations. As such, these models can result in tumors that arise in clinically relevant locations and resemble patient tumors histologically, but these models end up lacking the significant spatiotemporal, transcriptional, and genetic heterogeneity that is a hallmark of pHGGs. Due to this limitation, their efficacy in modeling a relevant clinical response, especially following primary treatment, is an inherent deficiency. This lack of heterogeneity can also be seen in differing responses to preliminary drug treatments applied to mouse models. Barton et al. [106] used two RCAS/tv-a-mediated mouse models of DMG, both driven by PDGF-B in *Nestin*+ cells, one of which was *Ink4a-ARF*-deficient and one that was *p53*-deficient. They found that a CDK4/6 inhibitor was effective at halting tumor formation in the *Ink4a-ARF*-deficient mouse but not in the *p53*-deficient mouse. While findings such as this could potentially have positive implications regarding the efficacy of individual drugs on certain tumor clones, evaluating treatment responses on models that are based on very specific mutations limits their clinical significance. Hence, given the inherent heterogeneous mixture of mutations found in patient tumors, evaluating treatment responses using GEMMs may be less clinically relevant than when using a patient-derived xenograft (PDX) model or patient tumor.

#### 3.2.2. Transplant-Based Models for pHGG

The lack of genetic complexity seen in GEMMs is valuable when answering biological questions about the pathogenesis of tumors arising from a specific subset of cells with a specific set of genetic alterations. However, PDX models are superior for investigating patient-derived tumor tissues, as they better represent the tumor microenvironment and clonal heterogeneity seen in patients [107]. By retaining the complexity of patient tumors, PDX are often considered a better predictor of patient response to potential therapeutics [108,109]. This section will describe the brief history of how PDX models have evolved in the study of pHGG, as well as current techniques and recent advances.

pHGGs were previously considered to be biologically similar to adult HGG, so early animal models often used other high-grade gliomas for injection into the brainstem [86,87,88,89,90]. Initial protocols developed specifically for modeling DMG in rodents used allograft via intracranial injection of adult rat glioma cell lines C6, F98, or 9L in both adult and neonate rats using a guide-screw system [86,87,88,89,90,110]. This method successfully established injection into the brainstem as a viable technique; however, findings on the pathophysiology of the disease were limited to the cell lines used, which were often of adult glioblastoma (GBM) origin and lacked heterogeneity reflected in tumor tissues. Current knowledge of the different molecular drivers in DMG compared to adult GBM underscores the importance of the use of relevant cell lines for the study of specific pediatric central nervous system tumors. This allograft method had shown potential therapeutic benefit for the chemotherapeutic agent used in GBM, temozolomide. Yet, temozolomide has not been efficacious for DMG in the clinic, demonstrating the limitations of using a model system that does not represent the correct molecular drivers of the disease [111].

Later models built on the early allograft models by creating bioluminescent PDX using the human GBM cell lines U87 MG, U251, GBM6, and GBM14 injected into the brainstem of athymic rats [91]. As with the allograft techniques, the animals treated with temozolomide showed a delay in tumor growth [91]. Additional studies performed similar experiments, engrafting GBM patient tumors obtained at biopsy or established GBM cell lines into immunocompromised mice [92]. Similar to the earlier allograft models of DMG, these models have established a protocol for the generation of tumors in the brainstem and provide a foundation to explore the tumorigenicity of DMG; however, they fail to properly represent the molecular characteristics of DMG.

As previously discussed, tissue collection of DMG samples has become increasingly common, and, as such, current xenograft DMG models primarily use patient tissues or established patient-derived cell lines. The first two studies to accomplish this used patient-derived tissue from a DMG tumor obtained at autopsy and used two methods of tissue preparation. An “indirect” method involved the culture of dissociated tissue cells prior to engraftment. A “direct” engraftment technique involved the injection of dissociated cells immediately after autopsy [51]. Both techniques resulted in tumors that histologically resembled DMG. Subsequently, variations of these protocols have become the standard for patient-derived xenografts of DMG [69,93].

Ongoing studies are investigating the use of neonatal mice instead of adult mice for xenograft studies due to the similar developmental time points between the neonatal mice and the children in which these tumors originate. The neonatal model presents implanted cells with the ability to crosstalk with a neurogenic environment and with the expectation that the transcriptional landscape of these tumors will more closely resemble those seen in patients. The developmental transcriptional landscape of patient tumors, in addition to the ages and locations in which they occur, strongly implies that they are arising from developmental/stem cell niches. Given the need for models that more accurately mimic the endogenous tumor microenvironment, in addition to models that will accurately represent treatment responses, neonates may offer a more representative model for pHGG progression in mice.

### 3.3. Organoid Models for pHGG

#### 3.3.1. Tumor Organoids

Organoid models of various organ types have been established as models for ex vivo tissue culture that accurately recapitulate the functional tissue structure of the representative organ. Similarly, tumor organoids are being increasingly used as an ex vivo model that better represents the clonal hierarchy than traditional in vitro cell culture without the high cost and time that murine models require. Tumor organoid models are derived from primary patient tumor tissues at the time of biopsy or post-mortem collection and are cultured using healthy tissue organoid-specific techniques, which vary depending on tissue type but usually result in dissociated tissue embedding in a 3D matrix [98]. The resulting culture is self-organizing, contains cell types with transcriptional heterogeneity, and more closely resembles the tumor obtained from the donor [95]. The potential to benefit patients, as a better representation of the disease in an in vitro environment with high throughput, could allow for therapeutic screens that are more accurate than traditional methods. This has shown to be variably efficacious, where patient tumor-derived organoids can be an indicator of drug response [95,96,97]. However, tumor organoid models lack the tumor microenvironment, including the vasculature and an immune milieu, which are important considerations for reliable tumor models.

At present, there have not been any notable strategies to develop complex pHGG tumor organoids; however, some investigators continue to incorrectly refer to any neurosphere-type culture as a tumor organoid model. There are some existing, similar models using GBM cells. One “GBM-on-a-chip” method has shown that culturing patient-derived GBM using a bioink composed of decellularized ECM results in a GBM organoid that is capable of invasion and accurately reproduces the resistance to therapeutics seen in the patient [94]. Another model has shown that orthotopic transplants of GBM organoids were histologically more similar than typical spheroid cultures [112].

#### 3.3.2. Organoid Co-Culture

One group has explored co-culturing organoids with both dorsal cortical and ventral forebrain organoids, in which a bespoke bioprinting assembloid model was used. This model was able to successfully fuse organoids with DMG cells grown in culture and reproduce prior results showing that the HDAC inhibitor panobinostat decreased the viability of DMG cells [101].

The generation of organotypic organoid models also allows for the co-culture of these models with cancer cells grown in vitro to model tumor invasion into adjacent healthy tissue. As with the tumor organoid models, GBM co-culture with cerebral organoids has been a recent focus in the literature. Studies have been performed showing that dissociated GBM cells were able to quickly invade and proliferate into cerebral organoids [99,100]. Considering the resemblance between cortical organoids and the developing brain and the neurodevelopmental origins of pHGG, co-culture methods will be a useful tool in the future, including high-throughput drug screens and functional genomic applications.

## 4. Concluding Remarks

We have provided a comprehensive review of the underlying biology of pHGG and the pre-clinical models being used, with a focus on the most prevalent form, DMG (formerly called DIPG). Work with these models has vastly increased our understanding of the molecular aberrations driving these cancers (Table 3). The halted progenitor state, initially identified by single cell RNA-sequencing, may provide options for differentiation therapy in the future, as demonstrated by the effects of Corin, an inhibitor of both HDAC and histone lysine-specific demethylase, to differentiate and reduce the tumorigenicity of DMG cell lines in vitro and in vivo [113]. In addition, we have reviewed the development of current pre-clinical models and their benefits for the study of DMG. Where applicable, the benefits and limitations of using specific pre-clinical models to answer biological questions have been described (Table 4), as well as the potential advantages of using emerging pre-clinical models. Given the developmental origins of pHGG, the development of tumor organoids and organoid co-cultures with existing and emerging cortical and brainstem organoid models holds promise as ex vivo methods for further understanding the pathobiology and developing novel therapeutic advances for this invariably fatal disease.

Given the dismal survival rate that any diagnosis of pHGG confers, the end goal of developing novel pre-clinical models for the study of pediatric high-grade glioma must be translated to the clinic. Current and improving model systems outlined here have described independent benefits and limitations and have aided in existing and emerging understanding of these diseases. Using the described techniques to move towards personalized therapy has already begun, and tumor biopsy followed by rapid cell culture and drug screening is now possible [114]. A move towards tumor organoid models and animal models that accurately represent patient tumor microenvironments may better represent these tumors and could prove to be more effective in the future, bridging a gap between the bench and bedside. The increase in available tissue, in concert with the increase in effective model systems, should allow for a more efficient pipeline in which potential therapeutics can be screened in a variety of patient-derived cell lines and/or organoids. Effective treatments can then be moved to animal systems that better predict patient outcomes, ultimately allowing for more effective drug discovery.

**Table 3:** Common mutations found in pHGGs.

Impact of H3.3K27M Mutation	Genes	Gene Function	References
Mutation: loss of function	* TP53 *	Tumor suppressor	[17,37,43,115]
Copy number abnormalities: amplification	* TOP3A *	Topoisomerase III alpha, involved in DNA replication	[30]
Copy number abnormalities: amplification	* CCND2 *	Cell cycle	[30]
Upregulation	*ATRX*	DNA recombination and repair	[30]
Downregulation	*CDKN2a (locus p16)*	Tumor suppressor	[41,116]
Mutation	*H3F3A*	Histone H3	[38]
NA	*PDGFRα*	Pathway involved in cell growth and division	[23]
NA	*FGFR1*	Proliferation of precursor cells	[115]
Copy number abnormalities: amplification	* MYC *	Proto-oncogene	[117]
Downregulation	*FOXG1*	Developmental transcription factor	[37]
Upregulation	*LIN28*	Neural precursor cell proliferation and differentiation	[23]
Upregulation	*PLAG1*	Neural precursor cell proliferation and differentiation	[23]
Upregulation	*IGF2BP2*	Neural precursor cell proliferation and differentiation	[23]
Impact of H3.1K27M mutation	Genes	Gene Function	References
NA	*ACRV1*	Bone regulation and skeletal development, heart development, and the reproductive system	[17,118,119,120]
NA	*EGOR*	Epigenetic regulator plays a role in noncanonical PRC1 and contributes to the specification of cell differentiation and development of body structure	[17,121,122,123]
NA	*PI3K*	Cell growth, proliferation, and migration	[17,124]

**Table 4:** Strengths and limitations of different pre-clinical models of pHGG.

Pre-Clinical Model	Benefits	Limitations
In vitro cell culture	Efficient to derive and maintain relative to animal models Greater availability Efficient for use in different forms of screening (i.e., CRISPR screening, drug screening)	Does not adequately represent the complexity of a tumor Homogeneity of clones Does not consider tumor microenvironmental conditions (i.e., hypoxia, ECM changes, angiogenesis, immune cell infiltration)
Genetically engineered mouse models (GEMMs)	Effective for understanding the origins of a tumor with a specific set of co-mutations Tumors usually arise in the spatially correct portion of the brain Given driver mutations, is sufficient to delineate the developmental origins of tumors Optogenetic models allow for the evaluation of neuronal circuit integration with tumor cells	Derived from a discreet number of pathogenic mutations; therefore, it is transcriptionally and genetically homogenous relative to the parent tumor
In vivo transplant models	Better represent patient tumor heterogeneity than in vitro and GEMM models Histologically, tumors usually resemble patient tumors Better predict patient responses to therapeutics	Findings on pathophysiology are limited to the patient cell lines used and are therefore inefficient compared to in vitro systems Requires an immunocompromised animal, thereby being unable to properly evaluate the immune landscape of the tumor microenvironment
Tumor organoid model	Better represents clonal hierarchy than in vitro systems Lower cost and time commitment than in vivo models Better high-throughput screening ability compared to other models	Lacks endogenous tumor microenvironmental features, such as vasculature and the immune system
Organoid-cancer co-culture model	Allows for analysis of interactions between cancer cells and a developing brain-like environment May allow for high-throughput screens that can assess brain toxicity	Most cortical organoid protocols are regionalized to a specific anatomical location of the brain and do not represent the entire brain microenvironment Lacks vasculature, immune milieu

## Data Availability

No new data were created or analyzed in this study. Data sharing is not applicable to this article.
